# Multidrug resistance found in extended-spectrum beta-lactamase-producing *Enterobacteriaceae* from rural water reservoirs in Guantao, China

**DOI:** 10.3389/fmicb.2015.00267

**Published:** 2015-03-31

**Authors:** Hongna Zhang, Yufa Zhou, Shuyuan Guo, Weishan Chang

**Affiliations:** ^1^College of Animal Science and Technology, Shandong Agricultural University, Taian, China; ^2^College of Animal Science and Technology, Shanxi Agricultural University, Taigu, China; ^3^Animal Husbandry Bureau of Daiyue District, Taian, China

**Keywords:** ESBL, *Enterobacteriaceae*, rural water reservoirs, multidrug resistance, *bla* genes

## Abstract

Extended-spectrum beta-lactamase (ESBL)-producing *Enterobacteriaceae* have been isolated from humans and animals across the world. However, data on prevalence of ESBL-producing *Enterobacteriaceae* from rural water reservoirs is limited. This study aimed to isolate and characterize ESBL-producing *Enterobacteriaceae* in rural water reservoirs in Guantao, China. ESBL-producing *Enterobacteriaceae* were found in 5 (16.7%) of 30 sampled rural water reservoirs. Sixty-six individual isolates expressing an ESBL phenotype were obtained in the present study. Species identification showed that 42 representatives of *Escherichia coli*, 17 *Klebsiella pneumoniae*, 4 *Raoultella planticola*, and 3 *Enterobacter cloacae*. Twenty isolates contained a single *bla* gene, including CTX-M (17 strains), TEM (2 strains), and SHV (1 strain). Forty-six isolates contained more than one type of beta-lactamase genes. ESBL-producing *Enterobacteriaceae* isolated in this study were all multidrug resistant. These findings indicated that the serious contamination of ESBL-producing *Enterobacteriaceae* in rural water reservoirs existed in Guantao, China.

## Introduction

The rational use of antibiotics helps control infectious diseases of humans and animals. Abuse and overuse of antibiotics in clinical practice has selected drug resistant bacteria and “superbugs” (Thompson et al., 2007; Yong et al., 2009; Pruden et al., 2012). Extended-spectrum beta-lactamases (ESBLs), resulting from amino acid substitutions in TEM-1, TEM-2, and SHV-1 enzymes were described in the 1980s and 1990s (Bush and Jacoby, 2010). ESBLs can hydrolyze penicillins, oxyimino-cephalosporins (e.g., cefotaxime, ceftazidime, ceftriaxone, cefuroxime, cefepime) and aztreonam but not cephamycins (e.g., cefoxitin, cefotetan) or carbapenems (Bush and Jacoby, 2010; El Salabi et al., 2013). ESBLs are predominantly found among *Enterobacteriaceae*, which are inhabitants of intestinal flora and important pathogens in nosocomial and community settings (Laurent et al., 2008; Azap et al., 2010; Song et al., 2011; Kang et al., 2013).

Extended-spectrum beta-lactamase-producing *Enterobacteriaceae* can spread between humans via contaminated food or water (Oteo et al., 2010; Piednoir et al., 2011) and acquire resistance to antibiotics by plasmids, transposons or other mobile vectors that carry resistance elements (Oteo et al., 2010; Peirano et al., 2012). Water environments are considered as important reservoirs for resistance genes (Gao et al., 2009), and maybe play an important role in transfer of drug-resistant genes between bacteria (Malakoff, 2002; Kummerer, 2004). More importantly, once ESBL-producing *Enterobacteriaceae* enter the intestine of humans and animals via drinking water, these bacteria could lead to the spread of resistance genes and to serious infections.

To date, numerous studies on ESBL-producing *Enterobacteriaceae* isolated from water environments have focused on wastewaters of hospitals and animal farms, and waters from rivers and lakes (Cabello et al., 2013; Varela and Manaia, 2013; Yang et al., 2013; Zurfluh et al., 2013; Haque et al., 2014). However, data on ESBL-producing *Enterobacteriaceae* isolated from drinking water in rural areas is very limited. In China, the main drinking sources for rural residents in many villages are water reservoirs. Therefore, the present study was conducted to describe the isolation and characterization of ESBL-producing *Enterobacteriaceae* in rural water reservoirs in Guantao, China.

## Materials and Methods

### Sampling Sites and Water Sample Collection

**FIGURE 1:**
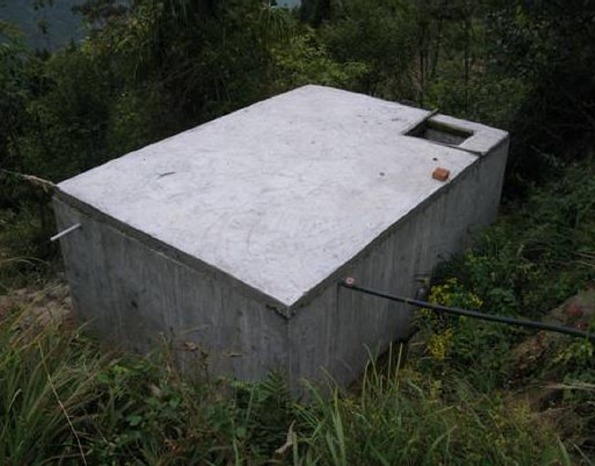
**The rural water reservoir commonly found in Guantao villages**.

**FIGURE 2:**
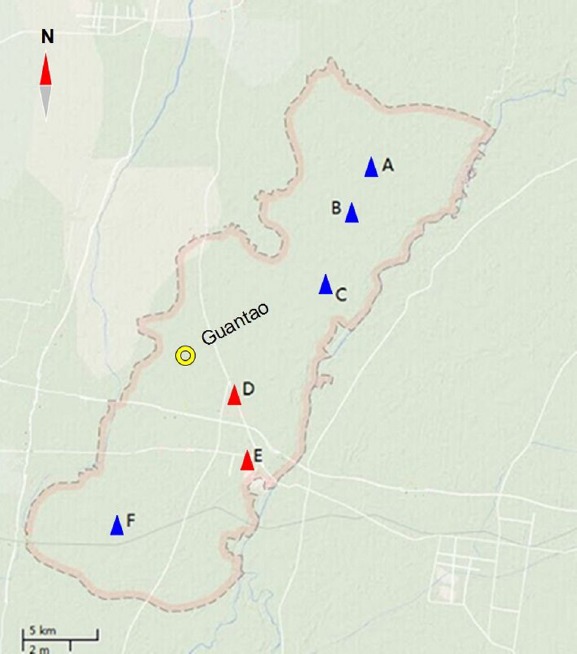
**Sampling sites.** Blue triangles **(A–C,F)** represent the communities where no ESBL-producing *Enterobacteriaceae* were detected in water samples. Red triangles **(D, E)** represent the communities where ESBL-producing *Enterobacteriaceae* were found in water samples. Yellow circle represents urban areas of Guantao County.

Between July and September of 2013, water sampling was conducted in Guantao, China (Figures 1 and 2). Five samples each were collected at six locations for a total of 30 samples. The water samples were collected from 50 cm below the water surface using sterile bottles (100 ml/bottle, one bottle/each reservoir). The collected water samples were stored on ice and immediately transported to our lab for further analyses within 3 h.

### Microbiological Analysis

Hundred milliliters of water was filtrated through a sterile 0.45 μm membrane (Millipore, Billerica, MA, USA), and then the filters were incubated in 20 ml of enterobacteria enrichment (EE) Broth (Becton Dickinson, Heidelberg, Germany) at 37°C for 24 h. One loopful of enrichment cultures was spread onto chromogenic Brilliance ESBL agar (Oxoid, Hampshire, UK) and incubated at 37°C for 24 h. The colonies with different color and morphology were picked and sub-cultured on sheep blood agar for 24 h at 37°C (Huang et al., 2010). Conventional biochemical methods and API ID 32 E (bioMérieux, Marcy l’Etoile, France) were used to identify the isolates. If isolates showed doubtful results, they were subjected to genetic identification based on sequencing of *rpoB* gene fragments (Mollet et al., 1997).

### Antimicrobial Susceptibility Testing and ESBL Confirmation

According to the protocols of the Clinical and Laboratory Standards Institute (CLSI, 2011), the disk diffusion method was used to test susceptibility of the isolates against 17 antimicrobial agents. The tested antibiotics were: ampicillin (AMP), cefaclor (CEC), cefazolin (CFZ), cefepime (FEP), cefotaxime (CTX), ceftazidime (CAZ), ceftriaxone (CRO), cefuroxime (CXM), aztreonam (AZT), ciprofloxacin (CIP), gentamicin (GEN), imipenem (IPM), ofloxacin (OFX), piperacillin (PIP), amikacin (AMK), chloramphenicol (CHL) and tetracycline (TET). According to the manufacturer’s protocols, Etest-ESBL strips (bioMérieux, Marcy l’Etoile, France) were used to confirm ESBL production. Isolates showing resistance to three or more antibiotic classes were defined as multidrug resistant (MDR). *E. coli* ATCC 25922 and *K. pneumoniae* ATCC 700603 were used as quality control strains.

### Polymerase Chain Reaction (PCR) to Detect *bla* Genes

The DNA of the isolates confirmed for producing ESBLs was extracted separately using a DNA extraction kit (Biospin plasmid extraction, Bioflux, Japan). According to previously published work, PCR was used to detect *bla*_TEM_, *bla*_CTX-M_, and *bla*_SHV_ genes using specific primers (Chen et al., 2010).

## Results

### Detection of ESBL-Producing *Enterobacteriaceae*

Extended-spectrum beta-lactamase-producing *Enterobacteriaceae* were detected in five water reservoirs of two rural communities (D: 2, E: 3). The five water reservoirs were all located close to chicken farms (approximately 12–15 m). No ESBL-producing *Enterobacteriaceae* were found in the other reservoirs, which were far away from rural villages and animal farms (approximately 1.0–1.5 km).

Sixty-six different isolates exhibiting an ESBL phenotype were obtained (D: 28, E: 38). The results of species identification showed that 42 *E. coli*, 17 *K. pneumoniae*, 4 *Raoultella planticola*, and 3 *Enterobacter cloacae* (Table 1).

**TABLE 1:** **Species composition of ESBL-producing *Enterobacteriaceae* from water samples**.

Species composition	Number	Percent (%)	Sources D/E
*E. coli*	42	63.6	18/24
*K. pneumoniae*	17	25.8	7/10
*Raoultella planticola*	4	6.1	2/2
*Enterobacter cloacae*	3	4.5	1/2
Total	66	100.0	28/38

### Antibiotic Susceptibility of ESBL-producing *Enterobacteriaceae*

Extended-spectrum beta-lactamase-producing *Enterobacteriaceae* isolates displayed similar drug-resistant trends. Nearly all ESBL-producing *Enterobacteriaceae* were resistant to the first- and second-generation cephalosporins (cefazolin, cefaclor, cefuroxime). These isolates were also resistant to the third-generation cephalosporins: cefotaxime (91.5%), ceftriaxone (67.9%), and ceftazidime (31.1%). Moreover, 48.1% of the isolates were resistant to cefepime (the fourth-generation cephalosporin), 51.9% to aztreonam (a monocyclic β-lactam antibiotic), and 89.6% to ampicillin.

**FIGURE 3:**
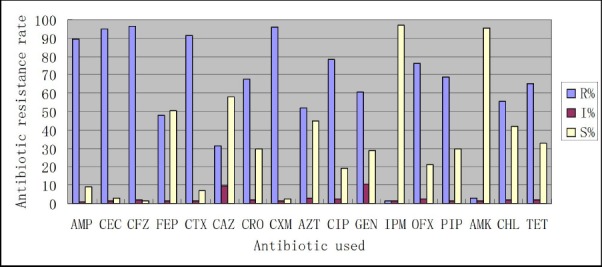
**Antibiotic resistance rates of ESBL-producing *Enterobacteriaceae.*** R: Resistance; I: intermediate; S: susceptible; AMP, ampicillin; CEC, cefaclor; CFZ, cefazolin; FEP, cefepime; CTX, cefotaxime; CAZ, ceftazidime; CRO, ceftriaxone; CXM, cefuroxime; AZT, aztreonam; CIP, ciprofloxacin; GEN, gentamicin; IPM, imipenem; OFX, ofloxacin; PIP, piperacillin; AMK, amikacin; CHL, chloramphenicol; TET, tetracycline.

Extended-spectrum beta-lactamase-producing *Enterobacteriaceae* were resistant to non-β-lactam antibiotics: resistant to ciprofloxacin (78.3%), gentamicin (60.4%), ofloxacin (76.4%), piperacillin (68.9%), chloramphenicol (55.7%), and tetracycline (65.1%). But the majority of ESBL-producing *Enterobacteriaceae* isolates were susceptible to amikacin (95.3%) and imipenem (97.2%) (Figure 3).

### Characterization of *bla* Genes in ESBL-producing *Enterobacteriaceae*

All 66 ESBL-producing *Enterobacteriaceae* carried *bla* genes. Among 66 ESBL-carriers, 20 strains carried only one *bla* gene (20/66, 30.3%), including 17 carrying *bla*_CTX-M_ (17/66, 25.8%), 2 isolates carrying *bla*_TEM_ (2/66, 3.0%), and 1 carrying *bla*_SHV_ (1/66, 1.5%). The other 46 isolates carried at least two *bla* genes (46/66, 69.7%), including 38 carrying *bla*_TEM_+_CTX-M_ (38/66, 57.6%), 6 carrying *bla*_SHV_+_CTX-M_ (6/66, 9.1%), and the other 2 carrying three *bla* genes (2/66, 3.0%) (Table 2).

**TABLE 2:** ***bla* gene types of ESBL-producing *Enterobacteriaceae* from water samples**.

*bla* gene types	Number	Percent (%)	Sources (D/E)
TEM	2	3.0	1/1
CTX-M	17	25.8	6/11
SHV	1	1.5	0/1
TEM+CTX-M	38	57.6	18/20
SHV+CTX-M	6	9.1	2/4
TEM+SHV+CTX-M	2	3.0	1/1
Total	66	100.0	28/38

## Discussion

In this study, ESBL-producing *Enterobacteriaceae* were detected in five out of 30 rural reservoirs (5/30, 16.7%), but not found in the other water reservoirs far away from villages and animal farms. This suggests that ESBL-producing *Enterobacteriaceae* are being shed into reservoirs from animal farms and anthropogenic activities.

Extended-spectrum beta-lactamase-producing *Enterobacteriaceae* isolates in this study were MDR. These isolates showed high resistance against the third-generation cephalosporins: cefotaxime (91.5%), and ceftriaxone (67.9%). Importantly, 48.1% of these isolates were resistant to cefepime, the fourth-generation cephalosporin. In addition, antibiotics resistance rates of these isolates to non-β-lactam antibiotics were also worrisome. But 95.3% and 97.2% of these isolates were respectively susceptible to amikacin and imipenem, which may be related with relatively low use of these medicines in this region.

In mainland China, previous investigations about ESBL-producing *Enterobacteriaceae* in water bodies and food-producing animals showed that *bla*_CTX-M_ gene was the dominant ESBL producer (Jin and Ling, 2006; Chen et al., 2010; Rao et al., 2014). Our data also identified *bla*_CTX-M_ gene as an important ESBL producer. Additionally, 46 out of 66 ESBL-producing *Enterobacteriaceae* isolates carried at least two *bla* genes and *bla*_CTX-M_+_TEM_ has become the dominant phenotype of ESBL, which was different from the previous result in clinical isolates (Zhang et al., 2014).

There were some limitations in this study: water sampling was carried out only in 30 rural water reservoirs, so the results may not be representative of the whole area; sequence analyses of *bla* genes encoding TEM, CTX-M, and SHV were not further conducted to identify variants of these types of enzymes; the risk factors associated with rural reservoir water carriage of ESBL-producing *Enterobacteriaceae* were not further analyzed.

In summary, these findings indicated that the contamination of ESBL-producing *Enterobacteriaceae* in rural water environments existed in Guantao, China, and the pollution may be closely related to local animal farms and anthropogenic activities.

### Conflict of Interest Statement

The authors declare that the research was conducted in the absence of any commercial or financial relationships that could be construed as a potential conflict of interest.
